# Health and Wellbeing in an Outdoor and Adventure Sports Context

**DOI:** 10.3390/sports8040050

**Published:** 2020-04-14

**Authors:** John Allan, Ashley Hardwell, Chris Kay, Suzanne Peacock, Melissa Hart, Michelle Dillon, Eric Brymer

**Affiliations:** 1 Carnegie School of Sport, Institute of Physical Activity, Leisure and Sport, Leeds Beckett University, Leeds LS6 3QS, UK; J.Allan@leedsbeckett.ac.uk (J.A.); A.G.Hardwell@leedsbeckett.ac.uk (A.H.); Chris.Kay@leedsbeckett.ac.uk (C.K.); s.peacock@leedsbeckett.ac.uk (S.P.); m.dillon@leedsbeckett.ac.uk (M.D.); 2 Carnegie Great Outdoors, Leeds Beckett University, Leeds LS6 3QS, UK; 3 Carnegie School of Education, Leeds Beckett University, Leeds LS6 3QS, UK; m.hart@leedsbeckett.ac.uk; 4 Discipline of Psychology, Australian College of Applied Psychology, Brisbane, QLD 4000, Australian

**Keywords:** wellbeing, outdoor and adventure sports

## Abstract

Outdoor and adventure sports (OAS) have been linked to positive health and wellbeing outcomes. This Special Edition brings together cutting-edge research and thought on the implications of this link. An analysis of the papers in this Special Edition reveals important insights into (i) the diverse and powerful outcomes derived from adventure experiences, (ii) how adventure experiences facilitate these outcomes, (iii) how best to design outdoor and adventure experiences. The evidence in this edition indicates a need for a more systematic approach to the inclusion of OAS as important to good health and wellbeing. OAS should be included as part of education, health, policy and planning.

## 1. Introduction

The World Health Organisation defines health as a “state of complete physical, mental, and social wellbeing, and not merely the absence of disease or infirmity.” [1] While in general, health is improving globally, there are still challenges. For example, in 2010, mental illness and substance abuse combined were the leading cause of non-fatal illness and the fifth leading cause of death and disease worldwide. In September 2015, the United Nations recognised mental health and wellbeing as priorities within the global development agenda. The natural environment has been presented as an important aspect of the global health improvement plan [2,3]. For instance, in 2017, the UK government published their 25-year environment plan [4] which emphasized helping people improve their health and wellbeing by using green space. The last two decades have been witness to a plethora of research from a vast array of fields, such as public health, ecology, geography, forestry, psychology, education, sport science and psychiatry, suggesting that physical activity in the presence of nature enhances health and wellbeing. For the most part, research has established that natural environments (i) enhance the impact of physical activity by increasing motivation, enabling emotional regulation, brain growth, recovery capability and protection from disease; and (ii) possess unique qualities unrelated to physical activity, such as restorative capabilities and stress-reduction which directly impact on health and wellbeing.

Outdoor and adventure sports (OAS) provide opportunities for generating physical and psychological benefits, whilst also delivering unique qualities unrelated to physical activity in nature. For example, immersion in nature provides the unique opportunity to relax and gain perspective on life. Furthermore, experiential learning processes have been linked to enhanced wellbeing outcomes beyond the impact of physical activity, nature and adventure. Programmed OAS are most often underpinned by an experiential learning framework combined with physical activity, immersion in nature and adventure. Therefore, OAS have the potential to directly impact on the health and wellbeing of participants and provide ideal interventions for mental health outcomes.

OAS differ from traditional notions of sport for a number of reasons. First, the term ‘sport’, often viewed as synonymous with structured competition, has a wider meaning which is reflective of the original etymological perspective. The English word ‘sport’, derived from old French word ‘desport’ originally referred to a ‘pastime’. This broad definition included the dimensions of self-development and recreation. This original notion means sports are considered to be multi-faceted, boundary-crossing activities, which do not necessarily involve structured competitive activity. Second, traditional sports are most often associated with structured rules and regulations allowing consistency in the competitive context and for measurement of what constitutes success. In most cases, OAS contexts are non-competitive and are therefore not bound by external rules and regulations. Furthermore, the notion of success in OAS is continually evolving and is not necessarily determined by factors such as speed, height, distance and so forth. Third, traditional sports most often take part in highly structured performance settings. Playing fields and sporting stadiums are marked out and measured to strict, often international, standards. OAS contexts are not defined in this way. The environment is invariably non-uniform and not structured to fit the sport; instead, the sport is often about adapting to environmental constraints.

While research on the outcomes of OAS has been growing over the last three decades, our understanding of how they enhance health and wellbeing still needs development. Traditional theoretical notions typically used to interpret findings are being questioned (Brymer, Davids, and Mallabon, 2014; Karmanov and Hamel, 2008; Keniger, Gaston, Irvine, and Fuller, 2013; Kjellgren and Buhrkall; Yeh et al., 2016). Research is also beginning to consider the importance of individual differences in OAS, such as feelings of connection to nature, and the person-environment relationship (Freeman, Akhurst, Bannigan and James, 2016; Freeman and Akhurst, 2015). Generally, research undertaken in OAS has focused on traditional activities such as walking and running undertaken in outdoor environments. However, outdoor and adventurous activities, from forest schools to extreme sports and beyond, are potentially more nuanced examples of physical activity in nature allowing focus on reconnecting people to the natural world. The articles in this submission use a wide-ranging spectrum of innovative methodologies to investigate the many issues concerning the impact of OAS on health and wellbeing, adding to our understanding of (i) the diverse and powerful outcomes derived from adventure experiences (e.g., Peacock et al.; Slee and Allan), (ii) how adventure experiences facilitate these outcomes (e.g., Hart; King et al.), and (iii) how best to design outdoor and adventure experiences (e.g., Schwenk; Shanahan et al.) if health and wellbeing is the program aim.

## 2. Outcomes from Outdoor and Adventure Experiences

Articles in this Special Edition highlight that health and wellbeing outcomes are available across multiple participant groups. Hart outlines how adventure facilitated a journey of self-awareness through the process of cognitive dissonance. Glover and Polley suggest that adventure contexts enhance the take up and adherence to physical activity, enabling the mental wellbeing of unfit young adults. Peacock, McKenna, Carless and Cooke demonstrate how adapted adventure has multiple benefits for the short-term recovery of military personnel. Their work with the Battle Back program facilitated by Carnegie Great Outdoors at Leeds Beckett University reports that mental wellbeing and self-determination outcomes were achieved far more rapidly than equivalent interventions. OAS programs were also beneficial for schoolchildren and university students, positively impacting on transitional stress, personal, social competencies and academic outcomes (Slee and Allan; Allan and McKenna).

## 3. How Adventure Experiences Facilitate the Outcomes

Submissions pointing to a deeper, more nuanced understanding of how adventure facilitates the positive outcomes warrant serious consideration. For example, the work by Hart surprisingly, and perhaps differently to other interventions, indicates that psychological discomfort and vulnerability triggered by adverse and difficult physical challenges might be an important stimulus for wellbeing outcomes. In a similar manner, the idea of adventure facilitating an embodied experience coupled with reflective processing was key to the therapeutic focus proposed by Schwenk. Further, it seems that the learning from adventure experience is directly attributable to individual capacities to continue to adapt to everyday life when interventions are applied to a military context (Kaiseler, Kay and McKenna).

## 4. How Best to Design Outdoor and Adventure Experiences

Designing adventure experiences to facilitate health and wellbeing outcomes requires different underlying principles than planning for other outcomes such as skill development. Various papers within this special edition point to a more informed understanding of the principles involved. One important message evidenced across a number of submissions (Shanahan et al.; Peacock et al.; Slee and Allan; Allan and McKenna; Kaiseler et al.; King et al.) is that intervention design must be deliberately intended to impact on everyday life, be developmentally appropriate, progressively adaptable and be evidence-based. A sound theoretical framework that justifies and supports this process is vital. Self-determination theory features prominently in many of the studies, providing a valid theoretical and applied framework for measuring and understanding intrinsic motivation in the adventure learning context. However, Shanahan et al. also provide an insight into a variety of potentially useful frameworks for nature-based health interventions depending on the intended outcome and the target beneficiaries. Underlying many of the papers, and most specifically highlighted by Farkic and Taylor’s slow adventure, was a call to design interventions that facilitated immersive, optimal, integrated and meaningful experiences rather than short, disconnected interventions. Webber and Hardwell add to this notion, showing how an integrated and embedded approach to adventure education within school curriculum is likely to have profound outcomes for learners and teachers. King et al.’s paper proposes an interactive structure that would be useful to program designers as a theoretical frame to guide the design of interventions intended to facilitate health and wellbeing outcomes.

Key points from this Special Edition need to be highlighted for the interest of program designers, policy makers and broader society:

- OAS are powerful facilitators of health and wellbeing outcomes. However, they become even more meaningful to people when deliberately designed for such outcomes.
- Unique aspects of OAS activities and programs exist, such as the role of discomfort, immersion in nature, progressive adaptability and physical challenge, that cannot be replicated by similar activities (for example traditional sports) which are directly linked to the development of enhanced health and wellbeing outcomes.
- The design of OAS programs needs to consider the intended outcomes, active ingredients of potential change, the group/individual characteristics, the environment and the activities. One size does not fit all, and it is not ideal to use generic ‘off the shelf’ program designs.
- Health and wellbeing outcomes from adventure experiences have a long-term positive impact on everyday life.

## 5. Conclusions

Interventions designed to enhance health and wellbeing are becoming more important. In recent years, OAS have been promoted as being ideal mediums for many health and wellbeing outcomes. This Special Edition highlights many of the broad opportunities OAS afford. Evidence presented in this edition supports the call for a more systematic approach to accepting OAS as important inclusions in the attempts to facilitate good health and wellbeing. OAS should be considered a fundamental part of the fabric of everyday life and included as part of education, health, policy and planning.
